# Successful pregnancy after mucinous cystic neoplasm with invasive carcinoma of the pancreas in a patient with polycystic ovarian syndrome: a case report

**DOI:** 10.1186/s13256-017-1343-y

**Published:** 2017-07-11

**Authors:** Conisha Holloman, S. J. Carlan, Lohini Sundharkrishnan, Angela Guzman, Mario Madruga

**Affiliations:** 1 Department of Obstetrics and Gynecology, Orlando Regional Healthcare, 1401 Lucerne Terrace, 2nd floor, Orlando, Fl 32806 USA; 2 Department of Pathology, Orlando Regional Healthcare, Orlando, FL USA; 3 Department of Medicine, Orlando Regional Healthcare, Orlando, FL USA

**Keywords:** Pregnancy, Pancreatic cancer, Polycystic ovarian syndrome, Whipple, Gemcitabine, Chemoradiation, Infertility, Mucinous cystic neoplasms

## Abstract

**Background:**

The incidence of invasive cancer within a mucinous cystic neoplasm of the pancreas varies between 6 and 36%. Polycystic ovarian syndrome is a disorder characterized by hyperandrogenism and anovulatory infertility. One surgical treatment that can restore endocrine balance and ovulation in polycystic ovarian syndrome is partial ovarian destruction. Successful pregnancies following preconception pancreaticoduodenectomies (Whipple procedures) and chemoradiation to treat pancreatic neoplasms have been reported rarely but none were diagnosed with pre-cancer polycystic ovarian syndrome-associated infertility. Gemcitabine is an antimetabolite drug used for the treatment of pancreatic cancer that can have profound detrimental effects on oogenesis and ovarian function. Whether the ovarian destructive property of gemcitabine could act as a method to restore ovulation potential in polycystic ovarian syndrome is unknown.

**Case presentation:**

A 40-year-old white American woman with a history of pancreatic cancer treatment with a Whipple procedure and chemoradiation with gemcitabine had a successful pregnancy after years of pre-cancerous anovulatory infertility and polycystic ovarian syndrome. She received no fertility agents and delivered full term via a spontaneous vaginal delivery with no pregnancy complications.

**Conclusion:**

Gemcitabine treatment for pancreatic cancer may result in resumption of ovulation in women with polycystic ovarian syndrome and these women should be counseled accordingly.

## Background

Mucinous cystic neoplasms (MCNs) of the pancreas comprise up to 25% of all resected cystic neoplasms [1, 2]. They originate in the pancreatic duct epithelium, are mostly slow growing, and almost exclusively occur in women [3]. The incidence of invasive carcinoma associated with MCN varies between 6 and 36% [4, 5]. The histologic features of the invasive component may be identical to typical ductal adenocarcinoma of the pancreas, but other types of invasive carcinoma have also been reported.

The usual surgical procedure performed for primary pancreatic malignancy is the Whipple procedure or pancreaticoduodenectomy [1]. Radiation, chemotherapy, or both (chemoradiation) may be added as an adjunctive in selected cases.

Polycystic ovarian syndrome (PCOS) is the most common endocrine disorder in reproductive-aged women and typically presents with insulin-resistance, hirsutism, and anovulatory infertility [6]. The incidence of PCOS is approximately 7% of reproductive-aged women and 51% of normogonadotrophic anovulatory women [7]. While there are a number of definitions of PCOS at least two of the three following elements must be present for a diagnosis: oligo/anovulation, hyperandrogenism, or polycystic ovaries on ultrasound. The etiology is largely unknown and treatment has included lifestyle modification [8], medications [9], and partial surgical destruction of the ovary [10] with wedge resection or ovarian drilling. The mechanism responsible for restoring ovulation after partial ovarian destruction is unknown.

Gemcitabine is a fluorinated nucleoside antimetabolite drug used for treatment of cancer of the pancreas and can have profound detrimental adverse effects on oogenesis [11]. A recent report suggests gemcitabine specifically is detrimental to preantral and antral ovarian follicles as well as non-luteinized granulosa cells [12].

Whether the ovarian disruption associated with gemcitabine can have effects on restoring ovulation similar to partial surgical destruction in PCOS is unknown.

We present a case of a woman with a history of 14 years of PCOS-induced anovulatory infertility and MCN-associated pancreatic cancer treated with a Whipple procedure, radiation, chemotherapy, and gemcitabine who had a successful conception and full-term delivery.

## Case presentation

**Fig. 1:**
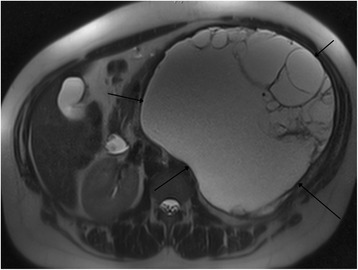
Magnetic resonance imaging of the abdomen showing large complex cyst between the *black arrows*

A 35-year-old gravida 0 white American woman with a 14-year history of unprotected intercourse without conception secondary to PCOS and 8 pack years of smoking presented to an emergency room with a lump in her left abdomen and sharp, left-sided, intermittent pain of 2 months’ duration. She was admitted and a computed tomography (CT) scan and magnetic resonance imaging (MRI) scan of her abdomen and pelvis showed an 18×19 cm left retroperitoneal mass (Fig. 1). Her body mass index (BMI) at admission was 29.9. She was discharged from the hospital and referred to a regional oncology service for further evaluation.

Two weeks later she underwent an endoscopic ultrasound that revealed the mass was compressing her stomach, pancreas, and esophagus. She had a fine-needle aspiration removing 60 milliliters of thick brown fluid with pathology showing only acute inflammatory cells. Staging CT scans were performed and there was no evidence of distant metastasis. A positron emission tomography (PET) scan displayed hypermetabolic activity at the periphery of the mass. Four weeks after her initial presentation, she underwent an exploratory laparotomy and decompression of the pancreatic mass yielding 2 liters of fluid. An attempt was made to resect the body of the pancreas but significant adhesions were noted and surgery was abandoned. The final pathology report was MCN and low grade dysplasia. A postoperative CT scan 2 weeks after her surgery showed the mass had increased in size to 20.3×15.1 cm with multiple septations. On a follow-up visit with her surgical oncologist her BMI was 29.1. At this point she was transferred to a centralized oncology center for surgical evaluation and management of the pancreatic mass with at least low grade dysplasia. Fourteen weeks after her initial presentation she underwent a Whipple procedure and splenectomy, removal of a retroperitoneal mass yielding 1.5 liters of mucinous material and an omental flap. Ovarian transposition was not performed. An 18 cm multiloculated cystic mass was present at the distal tip of the tail of her pancreas. Her final pathology revealed stage 1B invasive adenocarcinoma arising in the MCN with extensive high grade dysplasia (PTX N0 M0). All 37 lymph nodes were negative with negative venous or lymphatic invasion and the closest margin of 6 cm.

She returned to the regional cancer center and started single-agent gemcitabine 8 weeks postoperation. After four cycles, her post-chemotherapy CT scan showed no evidence of metastatic disease. She was started on concurrent fluorouracil (5FU) and targeted pancreatic radiation therapy 24 weeks after her definitive surgery and finished 25 fractions with minimal side effects and standard shielding. When she started her radiation her BMI was 32.4. Fifteen months after her initial presentation a quantitative beta-human chorionic gonadotropin (hCG) was obtained and returned 5783 mIU/ml. She had not changed sexual partners. An ultrasound revealed an intrauterine pregnancy with no embryonic pole or heartbeat. She had a spontaneous abortion at 6 weeks. There was no note of ovarian morphological abnormality. Her repeat CT scan 1 year later showed no evidence of pancreatic disease and she entered cancer surveillance at that time.

She first presented to our service 50 months after her initial presentation as a 40-year-old gravida 2-0-0-1-0 at 19 weeks’ gestation. She gave a history of an extensive infertility workup in the past prior to her cancer treatment which was consistent with PCOS-induced anovulation. Male factor workup was negative and she had attempted conception with the standard PCOS methods including timed intercourse after attempted medically induced ovulation without success. Weight loss did not result in conception. She did not attempt more invasive assisted reproductive techniques such as *in vitro* fertilization. Her targeted fetal ultrasound was normal as were serial obstetric scans. Her pregnancy was uneventful and standard prenatal testing was normal. She maintained normoglycemia on orally administered hypoglycemic agents. At 38 weeks, she had labor induced and delivered vaginally a healthy baby with Apgar score of 8/9 and birth weight of 2865 grams. She was discharged with her baby 2 days after her delivery.

## Discussion

This is the first report of a conception without the use of assisted reproductive technology and a successful term delivery in a woman with PCOS-induced anovulatory infertility after treatment with a Whipple procedure and chemoradiation. We performed a MEDLINE search of the English language literature and found two cases reporting preconception Whipple procedure [13, 14]. In neither case was conception achieved subsequent to chemotherapy, radiation exposure, and a pre-existing diagnosis of PCOS.

One of the suggested treatments of PCOS-induced anovulatory infertility is weight loss. However, our patient had no significant BMI changes and she had not even started an exercise program during her entire post-cancer preconception treatment so it is unlikely that any modification of lifestyle was operative in her sudden ovulation. In addition, she never took metformin which is an off-label but commonly used form of treatment for PCOS infertility. She conceived twice after taking four cycles of gemcitabine which is the only chemical she ingested that is known to be consistently toxic to gonads. It is unlikely that radiation was involved in her sudden resumption of ovulation since pelvic irradiation is known to result in diminished fertility potential [15].

There has been one report investigating molecular markers of human fertility related to chemotherapy involving gemcitabine indirectly. Chatzidarellis *et al*. [16] reported the effects of taxane-based chemotherapy on inhibin B and gonadotropins in males. Of their study participants, 60% were also on gemcitabine. They found a significant decrease in inhibin B, which represents Sertoli cell function, an increase in follicle-stimulating hormone (FSH) which is suppressed by inhibin B, and decrease in bilateral testicular volume. Furthermore, one animal study concluded that the anti-metabolite cancer drug gemcitabine was detrimental to preantral and antral follicles as well as mitotic non-luteinized granulosa cells [17]. Whether these data apply to females and ovarian changes is unknown but this is a suggestion that gemcitabine can, in fact, be associated with ovarian damage that may be consistent with the partial ovarian destruction that is a treatment method for PCOS-induced infertility [10]. Our patient’s unexpected resumption of ovulation and subsequent pregnancies could be unrelated to the effects of gemcitabine on her fertility axis but if there is a possible related relationship then reproductive-aged women with PCOS-induced infertility treated with gemcitabine should be aware that pregnancy is possible and proceed accordingly.

## Conclusions

In summary, women with PCOS-induced anovulatory infertility who are post-Whipple procedure and chemoradiation using gemcitabine should be counseled to use contraception if they do not desire pregnancy. Another option is a bilateral salpingectomy at the time of the Whipple procedure if they desire permanent sterilization.

This is a case of a pregnancy and term delivery following a Whipple procedure for IB adenocarcinoma of the pancreas discovered in a MCN in a 40-year-old woman. This case is very unusual because this patient had polycystic ovarian disease and anovulatory infertility for 14 years prior to her cancer. Her cancer was treated with surgery and chemoradiation. One of the chemotherapeutic agents used was gemcitabine which is known to have toxicity to ovaries. One of the treatments for PCOS infertility is partial ovarian destruction. Our patient resumed ovulation after her treatment. She had the same sexual partner, did not lose weight, and did not change her lifestyle. The important element to this case, in our opinion, is that women with PCOS who do not desire pregnancy and are scheduled for gemcitabine should be counseled about the possibility of resumption of ovulation.
